# Nothing about me without me: why an EU health literacy strategy embracing the role of citizens and patients is needed

**DOI:** 10.1186/s13690-019-0342-4

**Published:** 2019-04-02

**Authors:** Alexander Roediger, Kaisa Immonen-Charalambous, Markus Kujawa, Kristine Sørensen

**Affiliations:** 1 MSD International GmbH, Kriens, Switzerland; 2 European Patients‘ Forum (EPF), Brussels, Belgium; 3 Comité Permanent des Médecines Européens, Brussels, Belgium; 4 Global Health Literacy Academy, and Health Literacy Europe, Risskov, Denmark

**Keywords:** Health literacy, Citizens, Patients, Organisations, System

## Abstract

As a multi-faceted concept, health literacy concerns the capacities of people to meet the complex demands of health in a modern society, starting with basic skills and ending with active citizenship. The importance of advancing health literacy in Europe was recognised by the European Commission in various communications and initiatives and most recently by the OECD. However, a strategic approach combined with a long-term action plan is still missing. This commentary advocates for an EU strategy on health literacy to fully take into account the partnership of citizens and patients with professionals and decision-makers in health and health care to promote health literate societies.

## Background

As a multi-faceted concept, health literacy concerns the capacities of people to meet the complex demands of health in a modern society, starting with basic skills and ending with active citizenship [1]. Health literacy is linked to literacy and entails people’s knowledge, motivation and competencies to access, understand, appraise, and apply health information to make judgments and take decisions in everyday life concerning healthcare, disease prevention and health promotion to maintain or improve quality of life during the life course [2]. Health literacy is a skill in a wider context of public health including functional, communicative or interactive, and critical health literacy, ranging from personal health management to the shaping of social determinants of health [3]. Relying on elements of citizenship, health literacy relates to people-centred health where health policies are not developed “on behalf of” but “with” and “through” people who are in turn able to participate more fully and exert a higher degree of control over their health and wellbeing [1].

According to the World Health Organization in Europe, people with strong health literacy skills enjoy better health and well-being, while those with weaker skills tend to engage in riskier behaviour and have poorer health. Better health is directly linked to productivity, and indirectly to economic prosperity and wealth [4]. The importance of advancing health literacy in Europe was recognised by the European Commission in various communications and initiatives [5]. However, the fragmented approach may underestimate the potential and the role of health literacy in public health and health care of the future. This commentary advocates for an EU strategy on health literacy to fully take into account the partnership of citizens and patients with professionals and decision-makers in health and health care to promote health literate societies.

### Low health literacy – A large problem

Nearly half of all Europeans have inadequate and problematic health literacy skills. The European Health Literacy Survey revealed that 12% of all respondents have inadequate general health literacy and 35% have problematic health literacy. Limited health literacy in Europe is thus not just a problem of a minority of the population, in contrast, it is a public health challenge we cannot neglect [4].

Furthermore, limited health literacy is associated with higher cost. Research estimates that limited health literacy costs the American healthcare system US$ 73 billion per year and the Swiss healthcare system between CHF 1.5 and 2.3 billion per year posing a financial burden on health systems [4].

### Health literacy in the context of an ageing society

Future trends challenging health in the EU illustrate the need for a strategic approach to health literacy. Nearly one third of the population in the European Union will be 65 and older in 2060 [6]. Ageing is likely to lead to greater demand for older people which puts pressure on public budgets. They are more suscpetible to becoming ill and certain diseases appear mainly in older age such as cancer, which is now considered an aging-associated disease. At the same time, older age is associated with having limited health literacy. Limited health literacy may hamper access to prevention and health care services due to limitations in navigation, comprehension, and decision making [4].

### Health literacy, chronic conditions and new technologies

People with multiple conditions experience more problems with co-ordination and medical error [7]. According to the OECD [8], the multi-morbidity challenge requires a different approach, involving a shift from acute, episodic and hospital centric care to the management of chronic conditions, the delivery of continuity of care across different care settings and providers. Better co-ordination and continuity of care are important aspects of developing health literate organisations and systems considering active participation of people and citizens [4].

New technologies such as Personalised Medicine require new skills which are closely linked to the concept of health literacy as recognised in the Council Conclusions of the Luxembourg EU Presidency [9]. New technologies allow a prognosis of risk that was unthinkable a few years ago and lead to new levels of health- and health policy related decision-making for the individual but also health systems which directly touch on the concept of health literacy.

## Conclusion

Health literacy has been addressed in various EU initiatives, including the European Commission Strategy for Health, Conclusions of the Council of Ministers and in declarations [5]. Despite this strong recognition the approach is fragmented, there is no European strategy as in other policy fields, and regular assessment and monitoring systems of health literacy progress are not in place.

Recognising the magnitude of the health literacy challenge in Europe we need to bridge the gap to save time, save money and save lives [4].

Future developments in health require “health literate systems” which ensure navigation support and are readable for community members, consumers, and patients from all walks of life [4]. As important, health literacy is a critical skill to pursue an active health citizenship. Notably, health systems are shaped by society. The citizen as a member of the society plays a role by deciding on health-related legal, ethical and social questions, in the same way as Rousseau’s “contrat social” originated in the concept of the “citoyen” who decides about the laws and regulations to which he or she obeys [10].

Considering future health challenges, the human and financial impact, there is a need for a European Health Literacy strategy for a) assessing the role of citizens and patients in current policies, b) identifying gaps (regular surveys) and further potentials, and c) developing a health literacy action plan based on key recommendations. Essentially, if we agree on “Nothing about me without me” a dedicated health literacy strategy at EU level is needed.

## Abbreviations

EU: European Union
OECD: Organisation for Economic Co-operation and Development
WHO: World Health Organization
